# The Operation Theatre

**Published:** 1909-10-02

**Authors:** 


### PRACTICAL DEPARTMENTS.

### THE OPERATION THEATRE.

### I.—PAST AND PRESENT REQUIREMENTS.

IN no part of the modern hospital have there been more drastic changes than in the theatre and its appurtenances. Some forty years ago the large theatre with its tiers of seats or standing room for students, with possibly an adjoining room in which instruments were kept and cleaned, and in which the surgeons would wash their hands and change their coats, was all that was thought necessary. With the advent of antiseptic surgery the provision of sinks for washing sponges and of shelves for antiseptic lotions became necessary; a separate room for the administration of anæsthetics was required and apparatus for sterilising instruments were devised.

To-day the big theatre is a thing of the past, and in place of one large one, several smaller theatres are required, their number varying with the size of the hospital. Aseptic surgery has taken the place of antisepticism, and theatres with all these adjuncts have to be arranged to suit the altered conditions. Briefly the theory of aseptic conditions is that everything and everybody within the area set apart for operating must be sterilised. Surgeons, anæsthetists, assistants, and nurses wear sterilised garments, sterilised gloves, and cover their heads with sterilised wrappers, and some surgeons operate in masks. Everything brought into the theatre must first be sterilised, and in some theatres a visitor being shown over must don an overall and encase his boots within rubber boots. The structure of the theatre must be of such sort that it can be hosed down from top to bottom. The walls, the ceiling, and the floor must therefore be of some impermeable hard material, capable of being thoroughly cleaned in every part, and to facilitate the process of cleaning the floors should be laid to a fall with a carefully arranged outlet to a waste-pipe, the contents of which must ultimately be discharged into the drainage system. Just sufficient provision must be made for such a number of spectators as can profitably see what is going on; and this number is quite small. To carry out the system logically the stands for spectators should be approached from a separate passage outside the theatre, and separated from the theatre itself by a plate-glass screen.

Inside the theatre no fittings are permissible except possibly a sink for discharging septic material, one lavatory basin, and some glass shelves for things required during the progress of an operation. It is an open question whether the sink ought to be in the theatre at all, or whether it would not be better to send all septic matter out of the theatre immediately. And with regard to the lavatory basin, in one recent example a fixed basin with waste pipe was not allowed, a portable white metal basin being let into a metal ring supported on the wall. This basin is taken out and boiled in a steam steriliser after being used.

The modern theatre then is a comparatively small room, from which the array of sinks, washhand basins, and sterilisers which at one tim elined the walls have been rigidly banished. It follows therefore that room must be found outside the theatre for the many different apparatus required for washing and sterilising; and these rooms must be arranged with due regard to convenience of access to the theatre itself.

In a large hospital with a series of four or five theatres there will be a separate room for instruments provided with cases and sinks for washing instruments after use. The sterilising room must, of course, be within easy access of the theatres and will contain apparatus for sterilising the garments used by all concerned in an operation, bandages, and dressings, bowls, instruments, and every other appliance needed in the theatre. Two wash rooms will be needed, one for the surgeons and assistants, and one for the nurses. The latter will contain the sinks for washing porcelain vessels. In many instances a bath room is provided for the use of the surgeons; a cloak-room and w.c. should also be provided effectively cut off from the corridor into which the theatres open. A room for the administration of anæsthetics is needed in connection with each theatre, and space is required for a spare trolley to stand.

